# Hypercalcemic Crisis in a Patient with Post-Surgical Hypoparathyroidism

**DOI:** 10.1155/2019/3503651

**Published:** 2019-11-12

**Authors:** Wannita Tingsarat, Chalermchai Lertanasit, Jaruwan Kongkit, Thiti Snabboon

**Affiliations:** ^1^Department of Medicine, Faculty of Medicine, Chulalongkorn University, Bangkok, Thailand; ^2^Rayong Hospital, Rayong Province, Thailand; ^3^Surin Hospital, Surin Province, Thailand; ^4^Vachira Phuket Hospital, Phuket Province, Thailand; ^5^Excellence Center in Diabetes, Hormone and Metabolism, King Chulalongkorn Memorial Hospital, Thai Red Cross Society, Bangkok, Thailand

## Abstract

Calcium alkali syndrome (CAS), a relatively unusual etiology of hypercalcemia, is characterized by a classical triad of hypercalcemia, azotemia, and metabolic alkalosis. This condition has been described in patients who have taken an excess dose of calcium with an alkali or with a volume-depletion status. To diagnose CAS it requires a high index of suspicion and a detailed history of medication/supplement intake specifically for calcium-containing drugs and a history of all possible ingested alkali. We reported a case of post-surgical hypoparathyroidism whom later on was presented with hypercalcemic crisis due to CAS. The proposed mechanism of CAS and management are also included.

## 1. Introduction

Post-operative hypocalcemia from hypoparathyroidism is one of the common complications in patients who underwent thyroid or parathyroid surgery [1]. Calcium with vitamin D supplement is the standard treatment or prophylaxis regimen, which its complication is mild and self-limited [2]. We report a post-thyroidectomy patient with severe hypercalcemia from a presumptive diagnosis of calcium-alkali syndrome (CAS). The proposed mechanism and treatment are reviewed.

## 2. Case Report

A 61-year-old woman presented with symptoms of intractable nausea and vomiting for 10 days. Her past medical history included permanent hypothyroidism and hypoparathyroidism from subtotal thyroidectomy due to a huge multinodular goiter 2 years earlier. Her thyroid condition was well controlled with levothyroxine 600 *μ*g per week. She was also prescribed with 2,400 mg of elemental calcium and 1 *μ*g of alfacalcidol per day. On the last follow-up, 2 months prior to this visit, she was in good health with normal calcium and thyroid function levels. A thorough medical history review discovered that she had been taking different preparations of over-the-counter alfacalcidol, 0.25 *μ*g to 1 *μ*g/tablet for 6 weeks. She also increased the daily calcium supplement up to 4,800 mg of elemental calcium per day to control her tingling sensation. On physical examination, the patient was drowsy and moderately dehydrated. Her vital signs and neurological examination were unremarkable. Laboratory investigations showed profound hypercalcemia, mild hypophosphatemia and azotemia: serum calcium 17.08 mg/dL (8.5–10.5), phosphate 2.1 mg/dL (2.5–4.5), albumin 3.5 mg/dL and creatinine 2.0 mg/dL. Her venous pH was 7.46 and electrolytes showed mild hypokalemia and metabolic alkalosis: sodium 135 mEq/L potassium 3.4 mEq/L, chloride 95 mEq/L, and bicarbonate 29 mEq/L. Low levels of intact PTH 6.07 pg/mL (15–65), 25-OH vitamin D 19 ng/mL (>20) with a normal level of 1,25(OH)_2_ vitamin D 25.4 pg/mL (19.9–79.3) and normal levels of PTHrP were shown. Her thyroid function test was normal: FT_4_ 1.35 ng/dL (0.80–1.80) and TSH 2.52 *μ*IU/mL (0.35–4.10). The electrocardiography (ECG) showed sinus rhythm with normal QTc interval (410 ms). Her amylase and lipase levels were not elevated. The presumptive diagnosis of CAS was proposed from her triad of hypercalcemia, metabolic alkalosis, and renal insufficiency. She responded well to intravenous hydration with isotonic normal saline (4 L/d), subcutaneous calcitonin (200 IU q 8 h) and discontinuation of calcium and vitamin D supplements. Her clinical symptoms and serum calcium level returned to normal within 2 days of treatment. The patient was discharged on the 4^th^ day of hospitalization and was prescribed with 2,000 mg of calcium carbonate and 0.5 *μ*g of alfacalcidol daily. During the follow-up after 6 months, she did not have experienced episode of hypercalcemia and her renal function returned to baseline level.

## 3. Discussion

Severe hypercalcemia or hypercalcemic crisis may be accompanied by intractable nausea, vomiting, constipation, somnolence, coma, and sudden cardiac arrest. The etiologies of hypercalcemia are commonly caused by parathyroid, PTHrP or vitamin D-mediated mechanisms. Because of the availability and common usage of the over-the-counter calcium and vitamin D in clinical practice, the condition of CAS was reappearing [3, 4]. We report this unusual etiology of severe hypercalcemia in a patient with a history of high calcium-alkali (calcium carbonate) intake in combination with a high dose of vitamin D.

The key to the diagnosis of CAS depends on a history of ingestion of excess calcium (elemental calcium more than 4 g/d) with absorbable alkali or conditions which are prone to develop metabolic alkalosis, and exclusion of other causes of hypercalcemia [5]. Laboratory characteristics are hypercalcemia, normo-/hypophosphatemia, azotemia and metabolic alkalosis. Patients who appear to be at high risk for CAS include old age, renal insufficiency, volume depletion state, excessive vitamin D intake and medications that reduce renal function such as angiotensin-converting enzyme inhibitors (ACEIs), angiotensin receptor blockers (ARBs), NSAIDs, or thiazide diuretics [4, 5]. The onset of hypercalcemia may vary from days to months after receiving excess amount of calcium and alkali. In the past, this condition was described in the complications of peptic ulcer treatment due to the large dose of alkali or milk-containing regimen, so-called milk-alkali syndrome (MAS). After the introduction of H_2_-blockers and proton pump inhibitors, the prevalence of MAS started to decline. However, this condition has become common again after the awareness of osteoporosis and the easy access of calcium and vitamin D. Recently, CAS is reported as the third major cause of hypercalcemia among the hospitalized patients [3].

The exact pathogenesis of CAS is still elusive [4, 6]. When the affected patient received excess dietary calcium intake, hypercalcemia may develop from passive moving of calcium across the gut under the effect of vitamin D and gastric hyperacidity from its direct stimulation. Hypercalcemia also promotes diuresis by the blocking vasopressin effect in the collecting tubule (nephrogenic diabetes insipidus) and the stimulation of the calcium sensing receptors in the ascending loop of Henle. Its effect on renal arteriole vasoconstriction decreases the glomerular filtration rate (GFR) and increases the renal calcium reabsorption via the transient receptor potential vanilloid member 5 channels in the distal convoluted tubule. The metabolic alkalosis due to dehydration or vomiting increases tubular reabsorption of calcium which starts a self-perpetuating vicious cycle. In addition, hypervitaminosis D or vitamin D toxicity can also present as vomiting and may aggravate dehydration and alkalosis. The finding of normo-or hypophosphatemia or mild hyperphosphatemia (rare) may result from the phosphorus binding properties of calcium containing supplements [4]. The treatment includes adequate hydration and discontinuation of calcium-containing supplement. In case of severe or symptomatic hypercalcemia, calcitonin is administered to rapidly lower the calcium level in order to alleviate the symptoms. Bisphosphonate or dialysis is reserved in extreme cases of intractable severe hypercalcemia.

## 4. Conclusion

The awareness of this preventable condition should be raised in all patients whose protocol treatment contains a high dose of calcium and vitamin D supplement. We also emphasize the significance of a thorough history review particularly in self-medicating patients.
